# The impact of COVID-19 on maternal death and fetal death, a cohort study in Brazil

**DOI:** 10.1371/journal.pone.0290343

**Published:** 2023-08-17

**Authors:** Ana Paula Brioschi dos Santos, Creuza Rachel Vicente, João Paulo Cola, Luana Fiengo Tanaka, Juliana Rodrigues Tovar Garbin, Larissa Soares Dell’Antonio, Cristiano Soares da Silva Dell’Antonio, Angelica Espinosa Miranda

**Affiliations:** 1 Programa de Pós-Graduação em Saúde Coletiva, Universidade Federal do Espírito Santo, Vitória, ES, Brasil; 2 Departamento de Medicina, Programa de Pós-Graduação em Doenças Infecciosas, Universidade Federal do Espírito Santo, Vitória, ES, Brasil; 3 Department of Sport and Health Sciences, Technische Universität München, Munich, Germany; 4 Núcleo Especial de Vigilância Epidemiológica, Secretaria de Estado da Saúde do Espírito Santo, Vitória, ES, Brasil; UFSJ: Universidade Federal de Sao Joao del-Rei, BRAZIL

## Abstract

**Objective:**

The study aimed to evaluate the risk of maternal death and fetal death among pregnant women infected with SARS-CoV-2.

**Methods:**

This is a retrospective cohort study among pregnant women with secondary data from the National Live Births System (*Sistema Nacional de Nascidos Vivos*), National Mortality System (*Sistema Nacional de Mortalidade*), and e-SUS Health Surveillance System (*Sistema e-SUS Vigilância em Saúde*). Pregnant women confirmed for COVID-19 had positive RT-PCR between March 2020 and May 2021, pregnant women without COVID-19 were those without notification for disease. Maternal death, fetal death, and stillbirth were assessed as primary outcomes.

**Results:**

We included 68,673 pregnant women not notified as suspected of COVID-19 and 1,386 with a confirmed diagnosis of COVID-19. Among pregnant women with COVID-19, 1013 (73.0%) were aged 20 to 34 years, 655 (47.2%) were brown, 907 (65.4%) had ≥ 8 years of education, in the third trimester of pregnancy (41.5%), undergoing cesarean section (64.5%). In adjusted analyses, COVID-19 in pregnancy had a higher risk of maternal death (relative risk [RR] 18.73–95% confidence interval [95%CI] 11.07–31.69), fetal death/stillbirth (RR 1.96–95%CI 1.18–3.25), preterm birth [RR 1.18–95%CI 1.01–1.39], cesarean delivery (RR 1.07–95%CI 1.02–1.11), and cesarean delivery occurring before the onset of labor (RR 1.33–95%CI 1.23–1.44).

**Conclusion:**

COVID-19 may contribute to unfavorable pregnancy outcomes. Results showed that pregnant women infected with SARS-CoV-2 had a higher risk of maternal death, fetal death/stillbirth, preterm birth, cesarean delivery, and cesarean section occurring before the onset of labor.

## Introduction

Coronavirus disease (COVID-19), declared as a pandemic by the World Health Organization (WHO) in 2020, remains a major global public health problem, where about 670 million cases and 6.8 million deaths have been reported by February 2023 [1]. Pregnant and postpartum women were severely affected during the pandemic [2], although they were not initially considered a risk group for disease worsening [3–5]. However, they were found to be at a higher risk of intensive care unit admission, invasive ventilation, and death than non-pregnant women [6], as well as increased preterm births, growth restriction, miscarriage, fetal death, and maternal death [7–9].

The increase in maternal mortality during the pandemic has been mainly reported among low- and middle-income countries [10, 11] both due to the worsening of SARS-CoV-2 infection and the disruption of health services in the care of pregnant women [12]. In Brazil, between the years 2020 and 2021, the maternal mortality ratio increased by about five times compared to 2019. Between 2020 and December 2022, 24,203 cases of SARS-CoVID-19 were reported in pregnant and postpartum women with 2,043 deaths [13]. Vertical transmission of SARS-CoV-2 is rare and studies on postnatal complications in newborns are scarce [14, 15].

The early identification of signs of disease severity among pregnant or postpartum women allows the timely initiation of appropriate measures, such as supportive treatment, rapid referral, and admission to a ward bed or intensive care unit, according to institutional or national protocols. Furthermore, the identification of features that may lead to death becomes essential in pregnant and postpartum women with COVID-19 [4].

In this context, this study is justified by the substantial increase in maternal mortality during the pandemic period, and the important increase in hospitalizations of pregnant women due to respiratory distress [16]. Although it is known that the pandemic affected the health and welfare of pregnant women and their fetuses, which unbalanced the advances achieved over time, there are still few studies evaluating the effects of pandemic SARS-CVID-19 on gestational outcomes, especially in developing countries where the spread of SARS-CoV-2 contributes to worsening social inequalities and problems in the health care system [17]. Given the above, the objective of this study was to evaluate the risk of maternal death and fetal death among pregnant women infected with SARS-CoV-2.

## Material and methods

### Study design

This is a retrospective cohort study including data from pregnant women residing in the state of Espírito Santo, Brazil, this cohort of pregnant women is highly representative because all pregnant women entered into the national live births information system and the national mortality information system were enrolled. The study period was from January 2020 to January 2022 covering the period of the COVID-19 pandemic. The exposure assessed was SARS-CoV-2 infection during pregnancy, and groups with and without infection were compared. SARS-CoV-2 infection during pregnancy was confirmed by positive reverse transcriptase polymerase chain reaction (RT-PCR), this test was freely available to the entire population. The primary outcomes assessed were maternal death by COVID-19 and fetal death/stillbirth, and the secondary outcomes were preterm birth, cesarean delivery, 2-minute APGAR score less than 7, low birth weight, and cesarean delivery before the onset of labor.

### Study site

The state of Espírito Santo is located in the Southeast region of Brazil and has 78 municipalities and an estimated population of 4,018,650 inhabitants [18]. According to the ranking of the non-governmental organization Open Knowledge Brazil (OKBR), Espírito Santo was the most transparent state in the disclosure of data on COVID-19 in Brazil [19].

The beginning of vaccination against COVID-19 occurred in January 2021, but only in July, it started among pregnant and postpartum women without comorbidities. Given this, our sample comprised 88% of pregnant women without any dose of the vaccine among the group confirmed for COVID-19.

### Data source

Data on SARS-CoV-2 infection in pregnant women were accessed through the Health Surveillance System (e-SUS VS). Data on pregnancy outcomes were obtained from the National Live Births System (*Sistema Nacional de Nascidos Vivos*–SINASC) and National Mortality System (*Sistema Nacional de Mortalidade*—SIM).

The e-SUS VS system was implemented in the state of Espírito Santo in January 2020, replacing the Brazilian Ministry of Health’s Information System for Notifiable Diseases (*Sistema de Informação de Agravos de Notificação*—SINAN). It is the only official system for the compulsory notification of diseases in the state and was developed in partnership with the Pan American Health Organization (PAHO). In it, all notifications of cases and notifications of compulsory diseases seen in public and private health services are made. In Brazil, we use the term compulsory notification to define the mandatory communication to the health authority, carried out by doctors, health professionals, or those responsible for health establishments, public or private, about the occurrence of suspected or confirmed disease, injury, or health event public.

SINASC contains epidemiological information related to births reported in Brazil. Its data source is the Live Birth Certificate which is a document filled out in three copies, one of which is mandatory for birth registration at the notary’s office. Thus, it has universal coverage and representativeness due to its compulsory nature.

SIM is an information system that aims to collect comprehensive and reliable mortality data to support public health actions in various management spheres. The Death Certificate (DO) is the data source used by the SIM and should be filled out for all deaths, regardless of the place of occurrence. The physician is responsible for all the information in the DO. In our study, SIM was used to complement the information on mortality.

### Study variables

For evaluation of the outcome death in the exposed and unexposed group, information was taken from the mortality database (SIM), and for evaluation of the secondary outcomes the live birth database (SINASC) was used. From the e-SUS VS, variables referring to the clinical characteristics of pregnant women notified of COVID-19 were retrieved. They were gestational trimester of infection (first trimester, second trimester, and third trimester), comorbidities diagnosed in pregnant women with COVID-19 (lung diseases, cardiovascular diseases including hypertension, kidney diseases, smoking, obesity), and RT-PCR results (positive, negative, and not performed).

SIM was used for the variables referring to deaths. In maternal death, we followed the Tenth revision of the international classification of diseases and related health problems (ICD-10). We separated records with ICD-10 O as the base cause (ICD-10 referring to maternal mortality). We considered deaths by COVID-19 those records that had any of the ICD B 34.2 (coronavirus infection of unspecified location), B 97.2 (coronavirus, as the cause of diseases classified in other chapters) or U 07.1 (COVID-19, identified virus). Fetal death/stillbirth was defined as deaths under 27 days of age. For deaths due to COVID-19, we defined them as deaths registered under any part of the ICD B 34.2, B 97.2, or U 07.1.

Data regarding socio-demographic characteristics and secondary outcomes were taken from SINASC, such as age (less than 20 years, 20 to 34, above than 35), race/skin color (white, black, brown (*parda*), indigenous, and yellow), education (no education, up to 07 years of study, above 08 years of education), number of prenatal visits (up to 6 and more than 7 visits), gestational age at birth (up to 36 and above 37 weeks), type of delivery (cesarean or vaginal), 2-minute APGAR (from 0 to 6 or above 7), birth weight (up to 2499g and more than 2500g), and cesarean delivery before the onset of labor (yes or no).

Maternal age was determined based on gestation risk groups. Following the Brazilian Ministry of Health, up to six prenatal visits were considered. This recommendation aims to ensure the development of pregnancy and allow the delivery of a healthy newborn. We considered premature births with less than 37 weeks of gestation; APGAR scores lower than 7 were considered fetal distress; and low weight were the newborns with less than 2500g.

### Inclusion and exclusion criteria

The overall pregnant women residing in the state of Espírito Santo with delivery notified in SINASC between January 2020 and January 2022 were included. Pregnant women notified in the e-SUS VS for COVID-19 without laboratory confirmation by RT-PCR or with discarded diagnosis, multiple pregnancies, and duplicate registrations in the e-SUS VS were excluded. Some cases of pregnant women suspected of COVID-19 which had been recorded in the e-SUS were excluded from the sample when they were neither found in the SINASC nor any outcome found in the SIM, which resulted in their exclusion from the sample.

The exclusion criteria used aimed to reduce the bias between the exposed and unexposed group; therefore, pregnant women who underwent the RT-PCR test and obtained negative results were excluded from the sample. Although this test is the gold standard, we understand that a negative result cannot exclude the disease, and an inadequate discarding of the case may occur. Because it is an important risk factor in pregnancy, we excluded records with multiple pregnancies from both groups. Because it is an important risk factor in pregnancy, we excluded records with multiple pregnancies from both groups.

### Linkage

Initially, all pregnant women notified as a suspected case of COVID-19 between March 2020 and May 2021 (the period selected to follow up the outcomes until January 2022) were separated within the e-SUS VS. Then a probabilistic linkage of records between SINASC and e-SUS VS of the pregnant women was performed to obtain the pregnancy outcome when there was a live birth. The method described by Tanaka et al. in 2017 [20] was used. The process was conducted in three steps in the records, associated with a manual review of the doubtful pairs, seeking to classify them as true pairs or non-pairs, using blocking techniques. The blocking strategies contained a combination of the Soundex code of the first name and the Soundex code of the last name.

To calculate the weighted scores, we used the field “NAME” of the e-SUS VS and the field “NOMEMAE” of the SINASC which refers to the pregnant woman’s name. We also used the field “DTNASC” of the e-SUS VS and “DTNASCMAE” referring to the date of birth of the pregnant woman and when available we used the field "CODMUNRES" of the SINASC and “municipio_paciente” of the e-SUS VS referring to the municipality of residence of the pregnant woman. The clerical review was performed based on the cited fields in all the steps to improve the sensitivity relationship of the records. This process was conducted using the OpenRecLink software (version 2.9). All pregnant women with SINASC records that were not linked composed the population of pregnant women without COVID-19.

### Statistical methods

Absolute and relative frequencies were used for the descriptive analysis of the categorical variables. To check for significant differences in these variables between pregnant women with COVID-19 and pregnant women without COVID-19, Pearson’s chi-square test (χ^2^) was used.

For multivariate analysis, a Poisson model with robust variance was used. Initially, a crude model was run (Model A), followed by a model adjusted for maternal age (Model B). A third model was estimated and adjusted for maternal age and the secondary outcomes of the study (Model C). To evaluate the quality of the fits of each model, the Hosmer and Lemeshow test was used after each Poisson regression. In those situations, in which, for all models presented, the test statistic was not significant, when p>0.05, the adjustment was considered adequate. We considered maternal age a confounding factor requiring an adjustment in the model because maternal age is a factor associated with unfavorable perinatal outcomes in high-risk pregnancies [21].

The measure of association was the relative risk (RR) with a 95% confidence interval (95%CI) at 5% significance. Statistical analyses were performed in Stata v. 14.0 (StataCorp, CollegeStation, TX, USA).

### Ethical considerations

The study protocol was approved by the Research Ethics Committee of the Universidade Vila Velha—ES/UVV under opinion number 4,598,802. COVID-19 data are available via the following link: https://coronavirus.es.gov.br/painel-covid-19-es, SINASC (http://tabnet.datasus.gov.br/cgi/tabcgi.exe?sinasc/cnv/nvuf.def), SIM (http://tabnet.datasus.gov.br/cgi/deftohtm.exe?sim/cnv/obt10uf.def).

## Results

**Fig 1:**
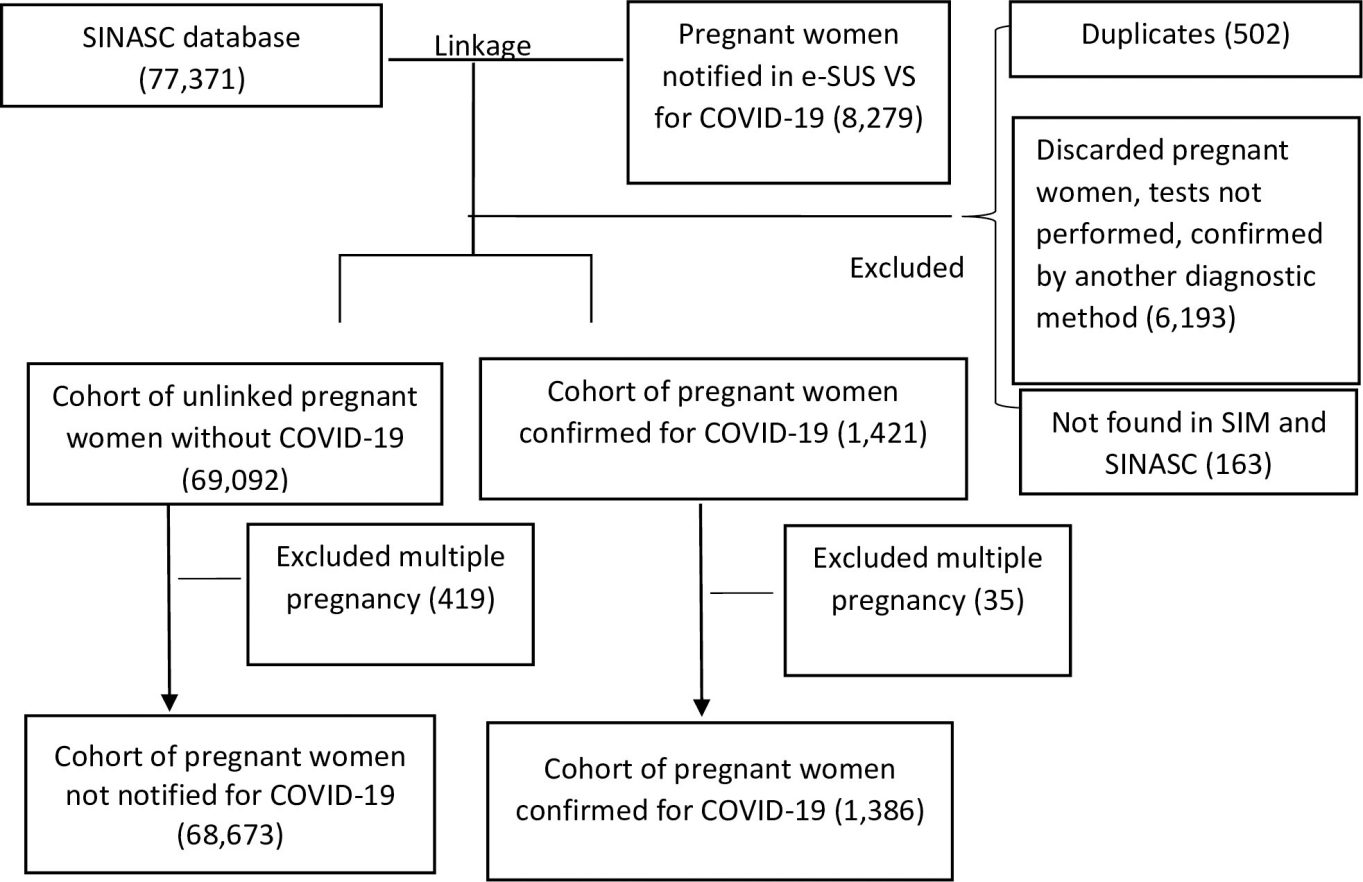
Sample constitution. SINASC—Live Births Information System (*Sistema de Informação de Nascidos Vivos*); e-SUS VS- e-SUS Health Surveillance System (*e-SUS VS- Sistema e-SUS Vigilância em Saúde*); SIM—Mortality Information System (*Sistema de Informação de Mortalidade*).

Between January 2020 and January 2022, SINASC registered 77,371 births, and 8,279 cases were found referring to pregnant women with suspected COVID-19. In these cases, the exclusion criteria were applied, and the SIM was consulted to verify the death outcome that was not present in SINASC, leaving 1,421 (17.16%) pregnant women confirmed for COVID-19 and 69,092 (89.3%) pregnant women not notified for COVID-19 (Fig 1).

Table 1 describes the distribution of the pregnant women’s sociodemographic variables, delivery characteristics, and the outcomes of pregnancy compared to whether or not COVID-19 infection was confirmed during pregnancy. Among pregnant women with COVID-19, 1013 (73.0%) were aged 20 to 34 years, 655 (47.2%) were brown, and 907 (65.4%) had ≥ 8 years of education. Regarding pregnancy characteristics among those positive for COVID-19, 576 (41.5%) became infected in the 3rd trimester of pregnancy, 1080 (77.9%) had 7 or more visits, and 894 (64.5%) had a cesarean delivery. Among the outcomes, there were 17 (1.2%) maternal deaths and 14 (1.01%) fetal deaths/stillbirths.

**Table 1:** Demographic and clinical characteristics of pregnant women confirmed for COVID-19 and not notified for COVID-19 in Espírito Santo, Brazil.

CHARACTERISTICS	N (%)	N (%)	P VALUE*
	PREGNANT WOMEN WITH COVID (1,386)	PREGNANT WOMEN WITHOUT COVID (68,673)	
**Age**			< 0.001
< 20	115 (8.3%)	9180 (13.37%)	
20 a 34	1013 (73.09%)	47640 (69.40%)	
> = 35	258 (18.61%)	11829 (17.23%)	
**Race**			< 0.001
White	402 (29%)	17776 (25.88%)	
Black	101 (7.29%)	5205 (7.58%)	
Brown / *Pardo*	655 (47.26%)	44696 (65.09%)	
Indigenous/ Yellow	91 (6.57%)	578 (0.84%)	
Unknown	137 (9.88%)	418 (0.61%)	
**Schooling**			< 0.001
Illiterate	1 (0.07%)	2206 (3.21%)	
Up to 7 years	136 (9.81%)	51428 (51.9%)	
≥ 8 years	907 (65.44%)	14533 (21.16%)	
Unknown	342 (24.68%)	506 (0.74%)	
**Gestational Trimester of Infection**			< 0.001
1st Trimester	262 (18.91%)	NA	
2nd Trimester	464 (33.5%)	NA	
3rd Trimester	576 (41.5%)	NA	
Unknown gestational age	84 (5.99%)	NA	
**Number of prenatal visits**			< 0.001
None	7 (0.51%)	695 (1.01%)	
Up to 6	285 (20.56%)	18578 (27.05%)	
≥ 7	1080 (77.92%)	48860 (71.15%)	
**Types of birth**			< 0.001
Vaginal	489 (35.28%)	27753 (40.41%)	
Cesarean	894 (64.5%)	40854 (59.49%)	
**Cesarean section occurred before the onset of labor**			< 0.001
No	948 (68.4%)	52844 (76.95%)	
Yes	438 (31.6%)	15829 (23.05%)	
**Gestational age**			0.036
Up to 36 weeks	137 (9.90%)	5703 (8.32%)	
Above 37 weeks	1247 (90.10%)	62824 (91.68%)	
**2-minute APGAR**			0.228
< 7	35 (2.53%)	1506 (2.19%)	
≥ 7	1338 (96.54%)	66742 (97.19%)	
**Birth weight**			0.310
< = 2500g	92 (6.64%)	4788 (6.97%)	
>2500g	1289 (93%)	63756 (92.84%)	
**Premature death**			< 0.001
No	1369 (98.77%)	68628 (99.93%)	
Yes	17 (1.23%)	45 (0.07%)	
**Fetal death/stillbirth**			0.068
No	1372 (98.99%)	68248 (99.38%)	
Yes	14 (1.01%)	425 (0.62%)	

* Pearson’s Chi-square test

Table 2 describes the distribution of comorbidities in pregnant women who had COVID-19 during pregnancy. The most frequent comorbidities were cardiovascular disease (4.8%), diabetes mellitus (3.2%), lung disease (2.0%), and obesity (1.4%).

**Table 2:** Presence of comorbidities in pregnant women with COVID-19 in Espírito Santo, Brazil (n = 1,386).

	N (%)
Comorbidities	PREGNANT WOMEN WITH COVID (1,386)
Lung disease	29 (2%)
Cardiovascular disease	67 (4.8%)
Kidney disease	2 (0.14%)
Smoking	10 (0.72%)
Obesity	20 (1.44%)
Diabetes	45 (3.24%)
Liver disease	1 (0.07%)
HIV	1 (0.07%)

Table 3 describes the relative risk of the primary and secondary outcomes for pregnant women who were confirmed for COVID-19 during pregnancy. In the crude analysis (Model A), pregnant women with COVID-19 at pregnancy had a higher risk of maternal death (RR 18.71–95%CI 10.74–32.61), preterm birth (RR 1.18–95%CI 1.01–1.39), cesarean delivery (RR 1.08–95%CI 1.04–1.12), and cesarean delivery before onset of labor (RR 1.37–95%CI 1.26–1.48). The age-adjusted analysis (model B) described a higher risk for fetal death (RR 1.72–95%CI 1.01–2.93) and maternal death (RR 18.19–95%CI 10.45–31.67). When the analysis was adjusted for age and secondary outcomes (Model C), there was an increased risk for both fetal death (RR 1.96–95%CI 1.18–3.25) and maternal death (RR 18.73–95%CI 11.07–31.69).

**Table 3:** Association between adverse outcomes among pregnant women with COVID-19 in Espírito Santo, Brazil.

	Pregnant women with COVID		Model A		Model B		Model C	
Variable	Yes	No	RR (95%CI)	P value	RR (95%CI)	P value	RR (95%CI)	P value
Fetal death/stillbirth	14 (1.01%)	425 (0.62%)	1.63 (0.96–2.77)	0.07	1.72 (1.01–2.93)	0.044	1.96 (1.18–3.25)	0.009
Maternal death	17 (1.23%)	45 (0.07%)	18.71 (10.74–32.61)	< 0.001	18.19 (10.45–31.67)	< 0.001	18.73 (11.07–31.69)	< 0.001
Preterm	137 (9.90%)	5703 (8.32%)	1.18 (1.01–1.39)	0.035	1.18 (1–1.38)	0.041	1.18 (1.-1.39)	0.041
Cesarean	894 (64.50%)	48856 (59.49%)	1.08 (1.04–1.12)	< 0.001	1.07 (1.02–1.11)	< 0.001	1.07 (1.04–1.12)	< 0.001
**APGAR < 7	35 (2.53%)	1506 (2.19%)	1.15 (0.82–1.60)	0.393	1.15 (0.82–1.60)	0.396	1.17 (0.83–1.61)	0.387
Birth weight < 2500g	92 (6.64%)	4788 (6.97%)	0.95 (0.78–1.16)	0.641	0.95 (0.78–1.17)	0.681	0.95 (0.78–1.18)	0.688
Cesarean section before the onset of labor OL*	438 (31.60%)	15829 (23.05%)	1.37 (1.26–1.48)	< 0.001	1.33 (1.23–1.44)	< 0.001	1.34 (1.21–1.46)	< 0.001

* Cesarean before the onset of labor

** APGAR <7 at the second minute of life

Model B: adjusted for maternal age.

Model C: adjusted by maternal age, preterm, cesarean section, and cesarean section before the onset of labor.

## Discussion

The study showed an increased risk of unfavorable pregnancy outcomes among those infected with SARS-CoV-2, such as maternal and fetal death/ stillbirth, preterm birth, cesarean delivery, and cesarean section before the onset of labor, when compared to pregnant women not notified for COVID-19.

Among the main findings in the comparative risk analysis, maternal mortality stands out, corroborating Brazilian and multinational cohort studies [22, 23]. Maternal mortality is known to be an indicator of the living conditions and health care of a population and also reflects the human development of a country, making it an important sensitive marker of the functioning of the health care system [24].

Regarding the sociodemographic profile, the group of pregnant women without COVID-19, under 20 years of age, non-white, and less educated, were the majority, revealing a feature of greater social vulnerability concerning the group of pregnant women with COVID-19. In parallel to these findings, studies have shown that in Brazil the less socially advantaged pregnant women are those of lower maternal age, who are not white, unemployed, and less educated [25]. These characteristics comprise a profile of health inequity present in the services, in which less favored women have less access to care and diagnosis of diseases, which can justify a lack of etiological diagnosis of SARS-CoV-2 [26].

It is known that quality prenatal care is essential for early detection, and even prevention, of pathologies, both maternal and fetal, and still favors the healthy development of the baby and reduces the risk of maternal-infant death. In Brazil, prenatal care should occur with at least six consultations [27]. We noticed that both groups reported having more than seven visits to the doctor, but more than 20% in each group had fewer visits than recommended.

The pandemic had a significant impact on prenatal care, restricting access and reducing follow-up visits, mainly due to fear of going to the health service [28]. However, pregnancy is an important risk factor for COVID-19 due to the greater susceptibility to alterations in the immune response that can result in the worsening of the disease [29], so the recommendation from the beginning of the pandemic was the need to ensure access to prenatal care to all pregnant women [30].

Cesarean section was the most common delivery route among the groups, as well as cesarean section occurring before the onset of labor. It is known that Brazil has 56.3% cesarean sections, the second highest rate in the world [31]. However, studies have reported an increased likelihood of cesarean section in pregnant women affected by COVID-19 [32, 33]. Savirón-Cornudella et al. (2021) [34] report that rates of preterm delivery and cesarean section are increased and influenced by different clinical practices. COVID-19 should not be an indication for early elective delivery unless, according to the Brazilian Federation of Gynecology and Obstetrics Associations (*Federação Brasileira das Associações de Ginecologia e Obstetrícia*—FEBRASGO), maternal or fetal decompensation occurs or when the pregnant woman evolves with severe or critical symptoms. The cesarean section offers risks, especially to women with limited access to adequate obstetric care [35].

Although COVID-19 is not an indication for cesarean section, the occurrence of this type before the onset of labor was higher in the group with COVID-19, which may be associated with the professional’s insecurity, increasing the number of cesarean deliveries in this group, due to the possibility of evolution to a severe condition, emergency cesarean delivery or premature delivery, besides the risk of maternal and neonatal death [36].

Most of the births occurred after 37 weeks of gestation in both groups, however, it was noticeable that preterm births were higher in the group among those with COVID-19. Although Brazil ranks 10th among countries with the most preterm births in the world [37], this finding corroborates the Centers for Disease Control and Prevention (CDC) of the United States, which demonstrated that COVID-19 among pregnant women increases the chance of preterm birth or gestational loss [38]. Another study conducted in the United States demonstrated that 12.9% of births among pregnant women with COVID-19 were premature (<37 weeks) compared to 10.2% reported in the general population in 2019 [39]. According to Agrawal L and Hirsch E (2012) [40], SARS-CoV-2 infection could trigger preterm labor via the toll-like receptor TLR-3.

In the present study, 67 pregnant women with COVID-19 (4.8%) had cardiovascular diseases, including hypertension. It is worth noting that factors such as being overweight, diabetes, hypertension, preexisting cardiovascular diseases, and chronic respiratory diseases increase the risk by almost four times to develop preeclampsia/eclampsia, reflecting an already known association between comorbidities and acute kidney damage in this public [41–43]. Similarly, data from the Surveillance for Emerging Threats to Mothers and Babies Network in the United States pointed to an association between more severe disease during pregnancy and older age, obesity, chronic lung disease, hypertension, and pregestational diabetes mellitus [43].

The risk of pregnancy complications, including preeclampsia/eclampsia/HELLP syndrome, is substantially increased in women with COVID-19, in addition to ICU admission, infections, preterm delivery, and low birth weight. A prospective cohort study showed a 22-fold increased risk of maternal mortality in this group compared to women not diagnosed with the disease [44].

A systematic review with meta-analysis similarly reported an increased risk of preeclampsia, preterm delivery, and stillbirth among pregnant women with SARS-CoV-2 infection than in those without SARS-CoV-2 infection [11]. In addition, the literature also points to increases in the number of stillbirths and maternal deaths, declines in maternal mental health, and an increased rate of ruptured ectopic pregnancies, raising a question about the delay in adequate care during the pandemic [45].

Although the relationship between the occurrence of fetal death/stillbirth and SARS-CoV-2 infection still shows inconclusive data in the literature, it is suggested that these complications may be indirectly linked to reduced mobility and exercise, increased exposure to smoking, increased caloric intake, poor glycemic control for pregestational and gestational diabetic women, poor supervision and control of hypertensive complications, and thyroid disorders during the pandemic confinement. Vertical transmission of the disease, which could be a risk factor, also needs further understanding since studies have shown limited evidence [45–47].

In Brazil, factors such as high birth rate, higher rate of cesarean sections, high maternal mortality rate, and prenatal care that faces chronic difficulties, associated with the absence of federal public actions aimed at mitigating the pandemic, contribute consubstantially to the findings presented [48]. Thus, monitoring pregnant women and knowing the behavior of COVID-19 and its effects on pregnancy is important to direct and implement specific prevention and control measures such as social isolation, use of masks in closed environments and with crowds, as well as encouraging vaccination, which is widely recommended by health authorities. The application of these measures, based on scientific evidence, aims to mitigate and reduce damage in this vulnerable group.

Due to the pragmatic nature of the data used, the study has the potential to describe the risk of outcomes in pregnant women infected with SARS-CoV-2 during pregnancy; however, there are limitations to be considered. Pregnant women discarded for COVID-19 and those who had diagnostic tests other than RT-PCR were excluded from the study, due to the possibility of false negatives, which may have created a selection bias in which potentially ill pregnant women were excluded, as well as the presence of SARS-CoV-2 infection in asymptomatic patients who were not tested. Nonetheless, the number of patients exposed overrides this bias as important differences were found between the groups consistent with other population-based cohort studies [49]. The presence of comorbidity is also an important confounding factor in the analysis of pregnancy outcomes, but, this information was not present in the unexposed group. As the study is an analysis of secondary data, there is an information bias when filling out the patient’s chart; however, the reliability of the data from the Brazilian Mortality and Live Births System and the fact that the variables used are objective diminish the existence of this bias [50].

## Conclusion

We conclude that COVID-19 may contribute to unfavorable pregnancy outcomes by demonstrating that pregnant women who were infected with SARS-CoV-2 had an increased risk of maternal and fetal death and stillbirth. In addition, among the undesirable effects verified, it was shown that pregnant women with COVID-19 were shown to have an increased risk of preterm deliveries, cesarean deliveries, and cesarean sections occurring before the onset of labor.

Maternal mortality and the unintended effects of SARS-CoV-2 infection in pregnancy have been widely studied worldwide. However, there are still uncertainties about the effect of COVID-19 on pregnant women and newborns, and more robust studies are needed to define the actual causality.
